# A Systematic Review and Meta-Analysis Comparing Liver Resection with the Rf-Based Device Habib™-4X with the Clamp-Crush Technique

**DOI:** 10.3390/cancers10110428

**Published:** 2018-11-08

**Authors:** Kumar Jayant, Mikael H. Sodergren, Isabella Reccia, Tomokazu Kusano, Dimitris Zacharoulis, Duncan Spalding, Madhava Pai, Long R. Jiao, Kai Wen Huang

**Affiliations:** 1 Department of Surgery and Cancer, Imperial College London, London W12 0NN, UK; m.sodergren@imperial.ac.uk (M.H.S.); isabella.reccia@gmail.com (I.R.); kpochitomo@yahoo.co.jp (T.K.); d.spalding@imperial.ac.uk (D.S.); madhava.pai04@imperial.ac.uk (M.P.); l.jiao@imperial.ac.uk (L.R.J.); 2 Department of General Surgery, University Hospital of Larissa, Mezourlo, 413 34 Larissa, Greece; zachadim@yahoo.com; 3 Department of Surgery and Hepatitis Research Center, National Taiwan University Hospital, Taipei 100, Taiwan

**Keywords:** liver cancer, liver resection, radiofrequency, Habib™-4X, clamp-crush technique

## Abstract

Liver cancer is the sixth most common cancer and third most common cause of cancer-related mortality. Presently, indications for liver resections for liver cancers are widening, but the response is varied owing to the multitude of factors including excess intraoperative bleeding, increased blood transfusion requirement, post-hepatectomy liver failure and morbidity. The advent of the radiofrequency energy-based bipolar device Habib™-4X has made bloodless hepatic resection possible. The radiofrequency-generated coagulative necrosis on normal liver parenchyma provides a firm underpinning for the bloodless liver resection. This meta-analysis was undertaken to analyse the available data on the clinical effectiveness or outcomes of liver resection with Habib™-4X in comparison to the clamp-crush technique. The RF-assisted device Habib™-4X is considered a safe and feasible modality for liver resection compared to the clamp-crush technique owing to the multitude of benefits and mounting clinical evidence supporting its role as a superior liver resection device. The most intriguing advantage of the RF-device is its ability to induce systemic and local immunomodulatory changes that further expand the boundaries of survival outcomes following liver resection.

## 1. Introduction

A central tenet to liver surgery lies in the complete oncological resection with minimal morbidity [1,2]. Advancement in liver resection techniques over the last few decades has resulted in improved morbidity, mortality and long-term survival. Consequently, the speciality of hepatobiliary surgery has experienced tremendous growth; however, liver resections are still contemplated as a high-risk surgical procedure with a mortality of ~5% and a morbidity of up to 40% [3,4,5]. Currently, liver resections are considered pertinent in the management of a wide variety of benign and malignant liver tumours including haemangiomas, colorectal liver metastases, hepatocellular carcinoma and hilar cholangiocarcinomas, with significantly reduced mortality in comparison to the earlier medical care available for such ailments and have added a significant number of productive years to patients’ life-span [6,7,8,9]. This improvement can be attributed to a number of factors including, increased use of parenchyma-sparing resections, lower intraoperative central venous pressure, better patient selection, ipsilateral portal vein embolization, staged resections in advanced diseases, the advent of newer devices for parenchymal transection, further improvements in perioperative patient management, and so forth [10,11,12,13,14].

Moreover, the development of non-surgical treatment modalities such as chemotherapy regimens, trans-arterial chemoembolisation (TACE), percutaneous radiofrequency ablation (RFA), microwave ablation, electroporation and cryotherapy could serve as an adjunct to the surgical management and produce a positive impact on the survival of liver cancer patients [15,16,17,18,19,20]. In particular, attention has turned towards liver resection owing to its ability to achieve oncological clearance; however, that comes with the price of several procedure-specific complications and increased perioperative morbidity, which may influence the disease-specific survival rates [21,22]. The foundation of liver resection surgery is the clamp-crush (CC) technique, which is regarded as a gold standard method of liver parenchymal transection, albeit that the post-resection outcomes are often limited by excessive bleeding, massive blood transfusions, bile leak and increased postoperative morbidity and mortality [23,24].

The advent of specialized liver resection devices with newer techniques for liver resection has introduced a new era in the surgical management of liver tumours [25,26]. Despite the continuous efforts to improve the surgical outcomes of liver resections, the intraoperative bleeding during liver parenchyma transection has remained a matter of prime concern and is often implicated in poor postoperative outcomes. The increased haemorrhage and blood transfusions have a negative implication of morbidity with increased bile leak, post-hepatectomy liver failure (PHLF), symptomatic collection, abscess, prolonged ICU stay, poor survival and mortality [27,28,29]. A vast majority of clinical studies have outlined the importance of limiting the intra-operative bleeding and blood transfusion in improving the morbidity and mortality of patients following hepatectomy [30,31,32,33,34].

Poon et al. demonstrated a significant decline in the morbidity and mortality from 37.0% to 30.0% and 7.5% to 3.7%, respectively, in a group of patients following hepatic resection with a median intra-operative blood loss of 750 mL and blood transfusion in 17% of cases, in contrast to a group with a median intra-operative blood loss of 1450 mL and blood transfusion in 68% of cases [35]. In addition, Yang et al. outlined increased intra-operative bleeding (≥800 mL) during hepatectomy as an independent risk factor of perioperative morbidity [36]. Furthermore, the present evidence suggests compromised oncological outcome and increased recurrence of hepatocellular carcinoma (HCC) in patients receiving blood transfusion perioperatively during hepatectomy [29,37]. The pre-eminence of decreasing perioperative blood loss and blood transfusion has been recognised, and different strategies have been implemented to limit the blood loss during parenchymal transection in liver surgery [38]. The hepatic vascular inflow occlusion technique or Pringle manoeuvre has been described, although it has limited applicability in patients with underlying liver disease secondary to heightened risk of ischemic reperfusion injury, incompetence to impede the back-flow bleed from hepatic veins and enhanced risk of ischaemic reperfusion (I/R) injury [39,40].

Radiofrequency (RF) energy can create an avascular plane for liver resection, this principle being first introduced by our group at Imperial College London, where later, a liver resection device, the bipolar Habib™-4X (Angiodynamics Inc., Latham, NY, USA), was developed [41]. Liver resection surgery has been transformed following the introduction of this device, which has facilitated the bloodless techniques of hepatic resection [41,42,43]. The radiofrequency-generated coagulative necrosis onto normal liver parenchyma creates a resection margin adjacent to the tumour. The coagulative necrosis helps in sealing of the blood vessels and bile ducts before transection of the parenchyma, hence limiting the requirement of the Pringle manoeuvre and blood transfusion [44,45]. The device is simple, cost effective, facilitates safe and efficient liver resection and fosters the practice of parenchymal sparing liver resections with a significant reduction in bleeding and subsequent perioperative complications [46,47]. To date, several retrospective and prospective (randomized or non-randomized studies) have been reported comparing various techniques of liver resections’ however, a meta-analysis comparing “bloodless technique of liver resection” (Habib™-4X) with the standard clamp-crush (CC) technique is lacking [48,49,50,51,52].

In spite of much literature available on liver resection, intriguingly, the quest to find the best technique and tool is still on. The present meta-analysis was undertaken to analyse the available data on the clinical effectiveness or outcomes following liver resection with Habib™-4X in comparison to the clamp-crush technique.

## 2. Materials and Methods

### 2.1. Search Strategy

We performed this meta-analysis following completion of registration (CRD42018085616) in PROSPERO, an international database of prospectively-registered systematic reviews. The search strategy was fundamentally designed according to the guidelines mentioned in the Cochrane Handbook for Systematic Reviews of Interventions and reported as per the guidelines proposed by the Meta-analysis of Observational Studies in Epidemiology [53,54].

A detailed literature search was completed on MEDLINE, EMBASE, Cochrane, Crossref, Scopus and clinical trial registries assessing the role of the RF-based device Habib™-4X in liver resection as an alternative to traditional the clamp-crushing (CC) technique. The search covered the period 2001 (the year of the first reported use of monopolar (Radionics Europe N.V., Wettdren, Belgium) and bipolar (Habib™-4X, Angiodynamics Inc., Latham, NY, USA) devices) to 4 July 2018 [41]. The medical subject headings (MeSH) ‘Liver resection’ OR ‘Radiofrequency device’ OR ‘Habib-4X’ ‘clamp-crush’ OR AND ‘Hepatocellular Carcinoma’ OR ‘Colorectal liver metastases’ OR ‘Cholangiocarcinoma’ OR ‘Hepatic metastasis’ were searched, adapting to each database without any limitation, to complete the analysis. The last search was completed on 10 September 2018. Further, all available conference abstracts, bibliographies and citation lists of the relevant articles were searched manually for additional studies.

### 2.2. Inclusion Criteria

The prospective or retrospective studies comparing the RF-based device (Habib™-4X) with the clamp-crush (CC group) technique of liver resections were selected for this meta-analysis. Further, all other available literature including editorials, reviews and letters were excluded. The primary outcomes of interest were blood loss, the requirement of blood transfusion and operative time. The secondary outcomes were adverse events, bile leak, post-hepatectomy liver failure, abdominal abscess, pleural effusion, overall hospital stay, morbidity and 30-day mortality (Table 1).

### 2.3. Data Extraction

**Figure 1:**
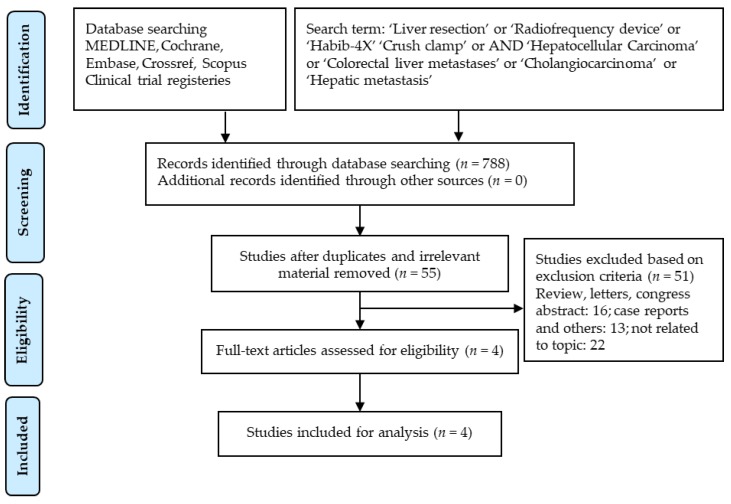
Search strategy and study selection used in this systematic review as per the PRISMA protocol.

The initial screening for the study was done independently by two separate physician reviewers, Kumar Jayant and Mikael H. Sodergren, who employed a two-stage method, the first stage involving scrutiny of titles and abstracts while excluding obviously ineligible studies. At the second stage, the full texts were considered in explicit detail to exclude ineligible studies. In the event of disagreement, disputes were resolved via consensus, and matters for which consensus could not be made were settled after much deliberation with the senior author NH. The complete search strategy and study selection were contemplated, performed and outlined according to the Preferred Reporting Items for Systematic Reviews and Meta-analyses (PRISMA) guidelines (Figure 1).

### 2.4. Statistical Analysis

The validity of pre-specified inclusion and exclusion criteria of the included studies was determined by using the Cochrane Risk of Bias tool. Each study was thoroughly analysed to evaluate the above-mentioned parameters (Table 2). The Cochrane Collaboration Review Manager (RevMan) Version 5.3 can analyse a minimum of two trials with the available continuous and dichotomous data. The effect measures used were mean difference (MD) for continuous data and odds ratio (OR) for dichotomous data, with 95% confidence intervals (CI). In the case of continuous data presented as median and range, the statistical methods described by Hozo et al. were applied to calculate the mean and standard deviation [55].

The heterogeneity (I^2^) between the trials was considered low with an I^2^ value ≤25%, moderate with an I^2^ value >25%, but <75% and higher with an I^2^ value of ≥75%. An I^2^ statistic of more than 30% was determined to be significant. In the stance of significant heterogeneity, the random effects model assessment was used following the evaluation of the forest plot while the fixed-effect model was applied in the situation of low heterogeneity [56,57]. Unfortunately, publication bias could not be assessed in the present study, as it requires at least 10 trials to assess it, and our current meta-analysis involved only four trials [58].

### 2.5. Surgical Technique

The liver resection techniques involving the RF-based device Habib™-4X have been outlined in our previous publication [59].

## 3. Results

### 3.1. Search Results

The primary literature search yielded a total of 788 manuscripts; of these, 784 articles were excluded following careful evaluation of the previously described selection criteria. After resolution of differences between reviewers, a total of four studies were retrieved for further review and data extraction [48,50,51,52]. These include three published papers on retrospective studies [48,50,52] and one with a prospective randomized study [51] (Table 2). The detailed data of all the studies related to the duration of surgeries’ adverse events, blood loss, requirement of blood transfusion, bile leak, post-hepatectomy liver failure, liver abscess, pleural effusion, length of hospital stay and 30-day mortality and are summarized in Table 2 and Table 3. All attributes of the analysed outcomes are structured further in this section.

### 3.2. Blood Loss and Quantity of Blood Transfused

**Figure 2:**
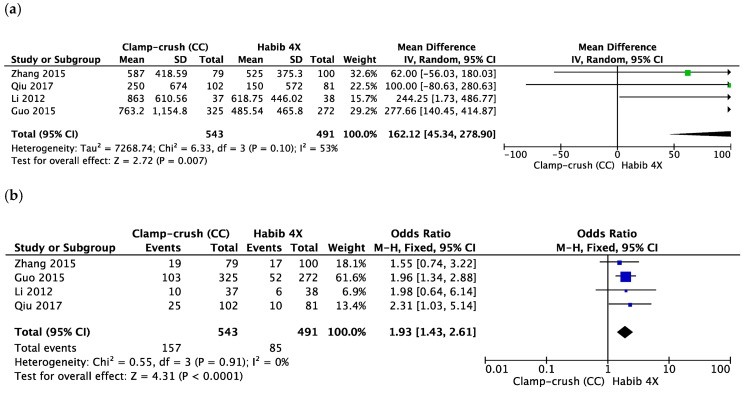
Forest plot representing the (**a**) total blood loss (mL), (**b**) number of patients requiring blood transfusion during liver resection comparing the control group (clamp-crush) with the study group (Habib™-4X). Squares’ size depicts the effects while comparing the weight of the study in the meta-analysis. The diamond shows the significant favour towards the study group (Habib™-4X) following the analysis. The 95 percent confidence interval is represented as horizontal bars.

The blood loss (mL) was significantly lower in the Habib™-4X group (MD = 162.12, 95% CI 45.34 to 278.90, *p* = 0.007, I^2^ = 53%) (Figure 2a). This outcome was determined by four studies [48,50,51,52], with a moderate heterogeneity between them. The Habib-4X and CC group included 491 and 543 patients, respectively.

Four studies reported the number of patients requiring blood transfusion in the two groups with low heterogeneity between the studies [48,50,51,52]. The Habib™-4X group received a statistically lesser amount of transfusion (MD = 1.93, 95% CI 1.43 to 2.61, *p* < 0.0001, I^2^ = 0%) (Figure 2b).

### 3.3. Operative Time

**Figure 3:**
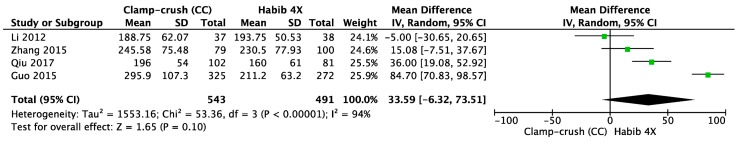
Forest plot representing the operative time (minutes) during liver resection comparing the control group (clamp-crush) with the study group (Habib™-4X). Squares’ size depicts effects while comparing the weight of the study in the meta-analysis. The diamond shows no favour towards any study group following the analysis. The 95 percent confidence interval is represented as horizontal bars.

Four studies reported operative time in minutes in the two groups with high heterogeneity between studies [48,50,51,52]. The random effects model revealed no statistical difference in terms of duration of surgery (MD = 33.59, 95% CI ™6.32 to 73.51, *p* = 0.10, I^2^ = 94%) (Figure 3).

### 3.4. Serious Adverse Events

**Figure 4:**
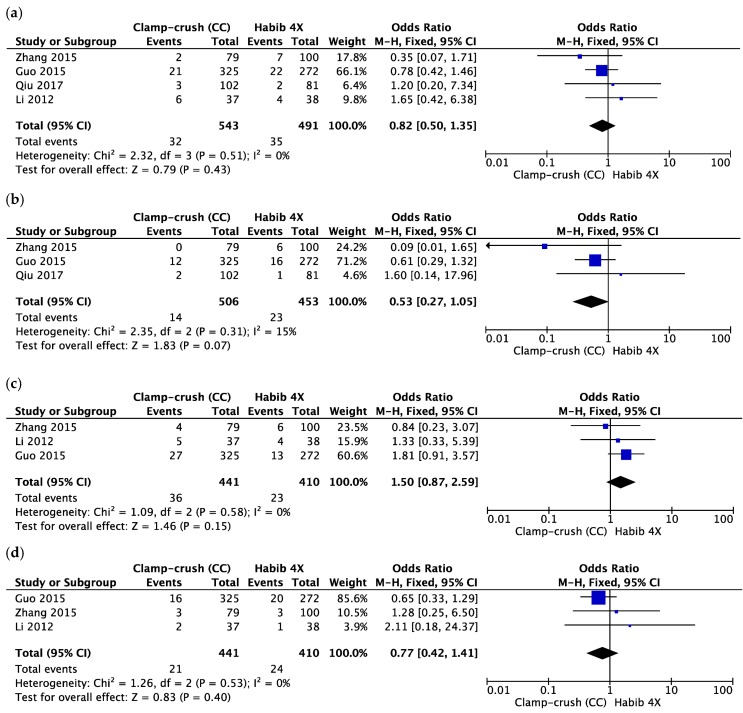
Forest plot representing the (**a**) bile leakage, (**b**) post-hepatectomy liver failure (PHLF), (**c**) pleural effusion and (**d**) abdominal abscess following liver resection comparing the control group (clamp-crush) with the study group (Habib™-4X). Squares’ size depicts effects while comparing the weight of the study in the meta-analysis. The diamond shows no favour towards any study group following analysis. The 95 percent confidence interval is represented as horizontal bars.

The rate of bile leakage was studied by four studies [48,50,51,52], with 543 patients in the CC group and 491 patients in the Habib™-4X group and a heterogeneity of 0%. The pooled data showed no difference between the groups (OR = 0.82, 95% CI 0.50 to 1.35, *p* = 0.43, I^2^ = 0%) (Figure 4a).

The PHLF incidence was reported in three of four included studies with low heterogeneity between them [48,50,51,52]. Both groups were statistically equivalent in term of given complications (OR = 0.53, 95% CI 0.27 to 1.05, *p* = 0.07, I^2^ = 15%) (Figure 4b).

We analysed pleural effusion rate given in three studies with low heterogeneity and found no difference between the Habib™-4X group and the CC group (OR = 1.50, 95% CI 0.87 to 2.59, *p* = 0.15, I^2^ = 0%) (Figure 4c). Similarly, there was no difference regarding abdominal abscess rate between them (OR = 0.77, 95% CI 0.42 to 1.41, *p* = 0.40, I^2^ = 0%). This outcome was reported by three studies with low heterogeneity between them (Figure 4d).

### 3.5. Length of Hospital Stay, Total Morbidity and 30-Day Mortality

The length of hospital stay was reported by four studies with low heterogeneity between them. In the fixed-effect model, there was no difference between the Habib™-4X group and the CC group (MD = 0.60, 95% CI −0.04 to 1.24, *p* = 0.07, I^2^ = 0%).

**Figure 5:**
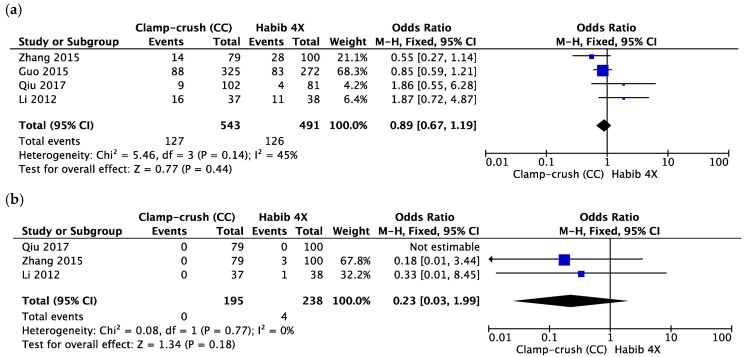
Forest plot representing (**a**) total morbidity and (**b**) 30-day mortality during liver resection comparing the control group (clamp-crush) with the study group (Habib™-4X). Squares’ size depicts effects while comparing the weight of the study in the meta-analysis. The diamond shows no favour towards any study group following analysis. The 95 percent confidence interval is represented as horizontal bars.

Three studies reported total morbidity, with 543 patients in the CC group and 491 patients in the Habib™-4X group. There was moderate heterogeneity between studies. The random effects model showed that both groups were comparable (OR = 0.89, 95% CI 0.67 to 1.19, *p* = 0.44, I^2^ = 45%) (Figure 5a). Similarly, there was no difference in terms of 30-day mortality between them (OR = 0.23, 95% CI 0.03 to 1.99, *p* = 0.18, I^2^ = 0%). This outcome was outlined by three studies with low heterogeneity between them (Figure 5b).

## 4. Discussion

Operative blood loss and blood transfusion are common complications of any surgical procedure; however, these are rather concerning in liver resection and are often associated with increased perioperative morbidity and mortality [29,37,60]. Intraoperative bleeding more commonly occurs during the hepatic parenchymal transection phase; hence, newer surgical modalities have been developed to target the real Achilles’ heel of liver resection and facilitate optimal transection with minimal blood loss [61]. Recognizing the vital role of minimizing the bleeding during liver resection, the “bloodless hepatectomy technique” was developed by Habib et al. [41]. Since, multiple publications have reported the benefit of this technique [48,49,59]; however, some researchers believe that the clamp-crush technique with inflow occlusion is still a reliable methodology of hepatic parenchymal transection [62,63,64,65,66,67].

The Pringle manoeuver and clamping of the hepatic pedicle is the most traditional and effective methodology to minimize blood loss during liver surgery [68,69,70,71], albeit the pathophysiological implications in the form of induction of liver ischaemia and ischaemic reperfusion injury are concerning and particularly unpredictable in patients with decreased hepatic reserve [62,72]. The intermittent vascular occlusion technique has been introduced as an alternative to lessen the degree of ischaemic injury to the liver parenchyma during hepatic resection [73]. Studies demonstrated equivalent bleeding control with similar or less deterioration in postoperative hepatic function and comparable operative time [74,75]; however, they did not result in a statistically-significant decrease in adverse event, morbidity, length of hospital stay and mortality, leading to a blunted enthusiasm to use this modality in hepatic resection. In addition, a meta-analysis by Rahbari et al. (2008) included eight randomized control trials containing 558 patients and concluded that the routine application of portal triad clamping does not add any benefit over no portal triad clamping and ought not to be practiced as a standard procedure [76].

In the presence of ongoing contention, over the safest and most efficient technique for liver resection, the present meta-analysis combines and quantifies the direct evidence present in studies and provides a systematic evaluation and statistical analysis of all the available outcomes with RF-based resection in comparison with the clamp-crush technique of hepatic resection. To our knowledge, this is the first meta-analysis comparing the outcomes of liver resection performed by the RF-based device Habib™-4X with the clamp-crush technique and eloquently outlines the broader picture of this practice with primary focus on blood loss and blood transfusion and subsequent complications. The data analysis was conducted using a rigorous methodology, which led to a sample size of 543 patients, who underwent liver resection with the CC technique, to 491 patients with the RF-based device Habib™-4X and demonstrated intriguing results. Further, in the discussion, we have highlighted our findings and the impact of both surgical modalities in liver resection.

Blood loss and blood transfusion have been frequently implicated in the increase in morbidity and mortality subsequent to hepatic resection. The pooled data of our meta-analysis not only demonstrated a significant reduction in the blood loss in the Habib™-4X group, but also showed the decreased requirement of blood transfusion. One of the major benefits of the Habib™-4X device is reduced blood loss without portal triad clamping, which is invariably often required in CC techniques and approximately one third of liver resections performed with the Cavitron ultrasonic surgical aspirator (CUSA) [26].

In an attempt to investigate the role of three different hepatic resection techniques: CC, CUSA and bipolar device (LigaSure), Doklestic et al. (2012) conducted a randomized clinical trial and demonstrated no differences in terms of intraoperative blood loss, blood transfusion, postoperative complications and mortality; however, all the patients involved in the trial had ischemic preconditioning and intermittent inflow occlusion [77]. Therefore, the decrease in operative blood loss and blood transfusion requirement observed here was considered secondary to inflow occlusion, which did not get translated into improved postoperative outcomes or reduced rate of complications. The plausible explanations for the observed findings could be ascribed to the higher degree of ischaemic insult to the hepatic tissue and increased risk of I/R, which may be more pronounced in livers with underlying disease such as cirrhosis or fatty changes [78,79,80]; however, this has no value in the case with the RF-based device Habib™-4X, as reduced blood loss observed during liver resection was owed to RF-induced coagulation of the liver tissue.

The postoperative infectious complications are considered as one of the important reasons for the morbidity and mortality observed following hepatic resection and remain as a matter of prime concern during recovery. Previous single centre’s experience reported increased rates of abdominal abscess following RF-based liver resection compared to the CC technique [81]. In contrast, Li et al. (2013) reported the incidence of abdominal abscess as 2.6% and 5.4% in the RF-Habib™-4X and CC group, respectively [51].

The review of the available literature in the present meta-analysis has demonstrated no difference in terms of the abdominal abscess in the compared groups. The data analysis of all the included studies in the index meta-analysis reported bile leak; however, no statistically-significant difference was observed in the present meta-analysis, which is in accordance with the reported incidence of bile leak of ~10% seen the literature.

Further, pooled data analysis outlined no statistically-significant differences in terms of PHLF, pleural effusion, hospital stay, total morbidity and mortality. The effectiveness of the RF-based device Habib™-4X has been demonstrated in several published papers, and the outcomes are remarkable with both cirrhotic and non-cirrhotic patients; the plausible explanations of these observed benefits are inscribed in the basic tenets on which this device was built. The RF-induced coagulation not only limits the blood loss and the requirement of blood transfusion, but also prevents any ischemic insult to the hepatic parenchyma.

The present meta-analysis has a few limitations, which need to be acknowledged, and caution ought to be exercised whilst interpreting these results, especially owing to the observed clinical heterogeneity between the included studies. The random effects model for pooled data analysis was used to limit the shadow of heterogeneity. Publication bias could not be excluded because of the limited number of included studies. Here, we could only identify four trials, and thus, further large-scale trials would provide much-needed data to allow firmer conclusions and elucidate the role of the RF-based device Habib™-4X in liver resection. Despite these limitations, this meta-analysis has outlined the safety and benefits of the Habib™-4X liver resection device in terms of reduced blood loss and decreased requirement of blood transfusion.

A recent study by Qiu et al. (2017) demonstrated better survival benefits following resection of liver tumour with the RF-based device Habib™-4X and outlined significant other benefits over the clamp-crush technique, notably due to minimal blood loss and less requirement of blood transfusion [48]. Similarly, Huang et al. (2017) compared the RF-based liver resection device Habib™-4X with CUSA and outlined significantly better disease-free survival [82]. The survival benefits observed in these studies could be a translation of better oncological outcomes associated with the interaction of the RF-assisted device with T-cells in the tumour microenvironment. The proliferative and energetically-dysregulated nature of tumour cells bring a metabolic dearth of the tumour microenvironment, which inflicts T-cells and creates “metabolic checkpoints” afflicting their endurance to survive, proliferate and function explicitly. The direct receptor-ligand interactions, expressing co-inhibitory ligands like programmed death-ligand 1 (PD-L1), inhibit CD8+ tumour-infiltrating lymphocytes’ function through programmed death-1 (PD-1). In addition, there are certain other checkpoint molecules in the tumour microenvironment, which negatively regulate T-cells’ functionality and are worth mentioning here, including cytotoxic T lymphocyte-associated protein-4 (CTLA-4), lymphocyte activating gene 3 (Lag3), mucin domain containing-3 (Tim-3), T-cell immunoglobulin and T-cell immunoreceptor with Ig. A “checkpoint inhibitor” such as anti-CTLA-4 and anti-PD/PD-L1 binds with these co-inhibitory checkpoint molecules and counter-inhibits the downregulation of T effector function, hence reinstating anti-tumour activity.

The exact reasons are not explicitly well defined, but could be explicable after the systemic and local immunomodulatory effect generated following the application of radiofrequency energy over tumour mass, causing T-cells’ infiltration into the tumour microenvironment [83].

The induced systemic antitumour immunity overcomes the challenges of micrometastases, which often escape destruction and are held responsible for the recurrence of hepatocellular carcinoma. The debris produced following RF-induced coagulative ablation during liver resection generated tumour antigens and chemokines, enticing the immunoprotective infiltrates, macrophages, neutrophils, dendritic cells (DCs) and NK cells. Dendritic cells activate the nuclear factor kappa-light-chain-enhancer of activated B-cells (NF-κβ) pathway, which stimulates CD8+ and CD4+ T-lymphocytes and promotes a systemic immune response also known as the “in-vivo dendritic cell vaccine effect” [84,85,86,87]. The increased understanding of the immunological behaviour of CD8+ T-cells has added a new perspective in the management advanced tumours and led to the development of newer drugs as checkpoint inhibitors, which augment the CD8+ T cells [88,89]. The potential effect is superlative as checkpoint inhibitors act in synergy with the RF-based device. Here, RF induces the infiltration of CD8+ T-cells at the resection margin, while checkpoint inhibitors augment the activity. In accordance, Duffy et al. (2017) conducted a study and demonstrated the activation of the immune system following introduction checkpoint inhibitors and the accumulation of intratumoral CD8+ T-cells after RF ablation, thus establishing the synergism of the combined use of checkpoint inhibitor tremelimumab and ablation in the management of advanced hepatocellular carcinomas [90,91]. The advantages of combining the checkpoint inhibitor with RF-energy continue to be unravelled. In particular, further knowledge and research are required to elucidate the effects induced through the combined application of RF-energy with immunotherapies that escalate the antitumour adaptive immune system exponentially. Nevertheless, the enhanced anticancer immune response exhibited through the synergism of RF-energy with immunotherapies has enormous potential for oncologic treatment for the coming years ahead.

## 5. Conclusions

**Figure 6:**
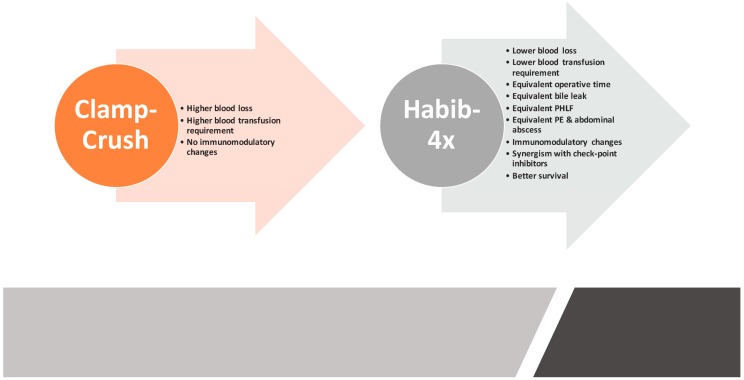
Comparative summary of the benefits of Habib™-4X based liver resection over the crush-camp technique. PHLF: Post hepatectomy liver failure; PE: Pleural effusion.

To recapitulate, the RF-assisted device Habib™-4X is considered a safe and feasible modality for liver resection in reference to the clamp-crush technique owing to a multitude of benefits and mounting clinical evidence supporting its role as a superior liver resection device (Figure 6). The most intriguing advantage of the RF-device is its ability to induce systemic and local immunomodulatory changes that further expand the boundaries of survival outcomes following liver resection. Furthermore, recent studies have outlined that the synergism of RF-energy and checkpoint inhibitors could have role in the management of advanced HCC tumours; however, further studies focusing on RF-energy and checkpoint inhibitors are required to ascertain the applicability of this combination.

## Figures and Tables

**Table 1:** Criteria for the inclusion of studies.

Study Design	Retrospective, Prospective, Randomized or Non-Randomized
Study group	Liver resection
Study size	Any
Length of follow-up	Any
Source	Peer-reviewed journals
Language	Any
Outcome measure	Primary: blood loss, blood transfusion, operative time; secondary: bile leak, post-hepatectomy liver failure, abdominal abscess, pleural effusion, overall hospital stay, morbidity and 30-day mortality

**Table 2:** Characteristics of studies included in the meta-analysis.

Study	Publication Year	Study Design	(Clamp-Crush) (CC) Group	Habib™-4X Group	Liver Disease	Operative Time (Minutes) (CC vs. Habib™-4X)
Li et al. [51]	2012	Randomized (Prospective)	37	38	HCC	188.7 ± 62.1 vs. 193.7 ± 50.5 (*p* = 0.28)
Guo et al. [52]	2015	Retrospective	325	272	HCC	295.9 ± 107.3 vs. 211.2 ± 63.2 (*p* = 0.00)
Zhang et al. [50]	2015	Retrospective	79	100	HCC	245.6 ± 75.5 vs. 230.5 ± 77.9 (*p* = 0.19)
Qiu et al. [48]	2017	Retrospective	102	81	HCC	196.0 ± 54.0 vs. 160 ± 61.0 (*p* = 0.00)

Abbreviations: CC, Clamp-crush technique; HCC: hepatocellular carcinoma.

**Table 3:** Post-hepatectomy analysis of outcomes in included studies.

Study	Blood Loss (mL) (CC vs. Habib™-4X)	Blood Transfusion (CC vs. Habib™-4X)	Bile Leak (CC vs. Habib™-4X)	PHLF (CC vs. Habib™-4X)	Pleural Effusion (CC vs. Habib™-4X)	Abdominal Abscess (CC vs. Habib™-4X)	Total Morbidity (CC vs. Habib™-4X)	Mortality 30 Days’ (CC vs. Habib™-4X)
Li et al. [51]	863.0 ± 610.5 vs. 618.7 ± 446.0 (*p* = 0.001)	10 vs. 6 (*p* = 0.23)	6 vs. 4 (*p* = 0.46)	NA	5 vs. 4 (*p* = 0.69)	2 vs. 1 (*p* = 0.54)	16 vs. 11 (*p* < 0.001)	0 vs. 1 (*p* = 0.321)
Guo et al. [52]	763.2 ± 1154.8 vs. 485.54 ± 465.8 (*p* = 0.003)	103 vs. 52 (*p* = 0.000)	21 vs. 22 (*p* = 0.44)	12 vs. 16 (*p* = 0.44)	27 vs. 13 (*p* = 0.40)	16 vs. 20 (*p* = 0.21)	88 vs. 83 (*p* = 0.35)	NA
Zhang et al. [50]	587.0 ± 418.6 vs. 525.0 ± 375.3 (*p* = 0.01)	19 vs. 17 (*p* = 0.24)	2 vs. 7 (*p* = 0.31)	0 vs. 6 (*p* = 0.04)	4 vs. 6 (*p* = 1.0)	3 vs. 3 (*p* = 1.0)	14 vs. 28 (*p* = 0.11)	0 vs. 3 (*p* = 0.23)
Qiu et al. [48]	250.0 ± 6 74.0 vs. 150.0 ± 572.0 (*p* = 0.005)	25 vs. 10 (*p* = 0.03)	3 vs. 2 (*p* = 0.89)	2 vs. 1 (*p* = 1.0)	NA	NA	9 vs. 4 (*p* = 0.39)	0 vs. 0 (*p* = 0.99)

Abbreviations: CC, clamp-crush technique; PHLF, post-hepatectomy liver failure; NA, not available.
